# Bioavailability Improvement of Carbamazepine via Oral Administration of Modified-Release Amorphous Solid Dispersions in Rats

**DOI:** 10.3390/pharmaceutics12111023

**Published:** 2020-10-26

**Authors:** Houli Li, Meimei Zhang, Lilong Xiong, Weiyi Feng, Robert O. Williams

**Affiliations:** 1 Department of Pharmacy, The First Affiliated Hospital of Xi’an Jiaotong University, 277 West Yanta Road, Xi’an 710061, China; lhl1416@xjtu.edu.cn (H.L.); fengweiyi@xjtu.edu.cn (W.F.); 2 Molecular Pharmaceutics and Drug Delivery, College of Pharmacy, The University of Texas at Austin, 2409 University Avenue, Austin, TX 78712, USA; zhmm03@hotmail.com; 3 School of Chemistry, Xi’an Jiaotong University, 28 Xianning West Road, Xi’an 710049, China; lilongxiong@xjtu.edu.cn

**Keywords:** carbamazepine, amorphous solid dispersions, modified-release, oral administration, bioavailability, thin film freezing

## Abstract

The purpose of this study was to improve the bioavailability of carbamazepine (CBZ), a poorly water-soluble antiepileptic drug, via modified-release amorphous solid dispersions (mr-ASD) by a thin film freezing (TFF) process. Three types of CBZ-mr-ASD with immediate-, delayed-, and controlled-release properties were successfully prepared with HPMC E3 (hydrophilic), L100-55 (enteric), and cellulose acetate (CA, lipophilic), defined as CBZ-ir-ASD, CBZ-dr-ASD, and CBZ-cr-ASD, respectively. A dry granulation method was used to prepare CBZ-mr-ASD capsule formulations. Various characterization techniques were applied to evaluate the physicochemical properties of CBZ-mr-ASD and the related capsules. The drug remained in an amorphous state when encapsulated within CBZ-mr-ASD, and the capsule formulation progress did not affect the performance of the dispersions. In dissolution tests, the preparations and the corresponding dosage forms similarly showed typical immediate-, delayed-, and controlled-release properties depending on the solubility of the polymers. Moreover, single-dose 24 h pharmacokinetic studies in rats indicated that CBZ-mr-ASD significantly enhanced the oral absorption of CBZ compared to that of crude CBZ. Increased oral absorption of CBZ was observed, especially in the CBZ-dr-ASD formulation, which showed a better pharmacokinetic profile than that of crude CBZ with 2.63- and 3.17-fold improved bioavailability of the drug and its main active metabolite carbamazepine 10,11-epoxide (CBZ-E).

## 1. Introduction

The gastrointestinal (GI) tract is the most convenient and popular route used for drug administration in the clinic, with better patient compliance and fewer costs [1]. Most marketed drugs are available in oral dosage forms. The dissolution of these drugs in GI fluids is a prerequisite for GI absorption and clinical efficacy. However, approximately 40% of drugs on the market and up to 70% of new chemical entities for drug development exhibit poor water solubility [2,3]. This poor water solubility results in additional problems. A low drug dissolution rate will limit drug bioavailability after oral administration, especially for biopharmaceutical classification system class II (BCS II) drugs, which have poor water solubility and high permeability [4,5]. Therefore, enhancement of the dissolution rate plays a crucial role in improving the oral bioavailability of BCS II drugs.

Numerous solubility-enabling approaches have been applied to improve the dissolution rate of BCS II drugs, including the use of cyclodextrins [6,7], cocrystal systems [8,9,10,11], nanoemulsions [12] or microemulsions [13], cosolvents [14], micelles [15], nanoparticles [16], nanostructured lipid carriers [17], amorphous solid dispersions (ASD) [18,19,20,21], coamorphous systems [22], etc. Among these approaches, ASD is one of the most reasonable strategies and has received increasing attention in recent years. ASD enhances the drug dissolution rate by improving its energetic state through disruption of the lattice structure [23,24]. Since drug absorption is a function of solubility and permeability [4], a significant advantage of ASD is that the preparation can increase the apparent solubility without a simultaneous decrease in membrane permeability [25,26]. Currently, many techniques have been applied to prepare ASD based on different mechanisms, such as spray drying, ball milling, hot-melt extrusion (HME), supercritical fluid technology, thin film freezing (TFF), Kinetisol^®^, and electrostatic spinning [27,28,29,30,31,32]. Drug-processing technologies should be selected according to the physicochemical properties of the drug, including its thermodynamic properties, miscibility and solubility with excipients, intermolecular interactions, etc. TFF is a typical particle engineering technology for developing ASD with an ultrarapid freezing rate [33]. Due to the high rate of supercooling, potential phase separation and drug nucleation can be successfully prevented, and ASD containing drug substances and excipients can be produced [31,34]. The ASD produced by TFF have large specific surface areas, which improve the dissolution rate of poorly water-soluble drugs and further enhance the oral bioavailability of these drugs in vivo.

However, most research on ASD has focused on immediate-release formulations, supersaturation, and stability [3]. Only a few studies of ASD with modified-release properties have been reported [35,36,37]. Compared to ASD with immediate-release properties, ASD with modified-release properties (delayed-release or controlled-release) can minimize the necessary dosage and frequency of administration, avoid toxicity, reduce side effects, and significantly improve bioavailability [35,38]. There are also some particular tradeoffs and challenges upon applying these formulations, including recrystallization in vivo, difficulty of drug release due to drug–polymer gelling, undesired supersaturation with a slow dissolution rate, etc. [35]. Hence, an in-depth study is needed for modified-release ASD.

**Figure 1:**
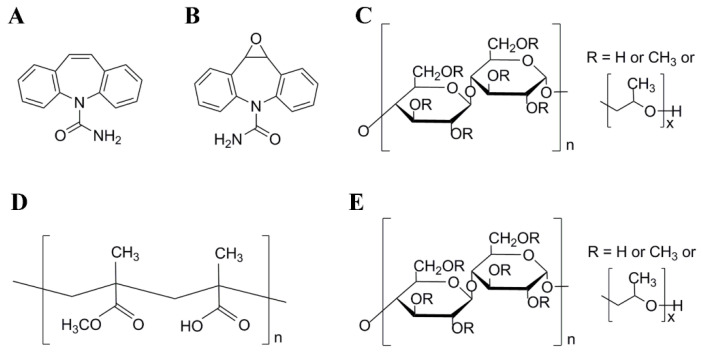
Chemical structures of CBZ (**A**), CBZ-E (**B**), HPMC E3 (**C**), L100-55, (**D**) and CA (**E**).

Carbamazepine (CBZ, Figure 1A), as a commonly prescribed antiepileptic drug, is widely used in the treatment of various forms of epilepsy in the clinic. In recent years, new antiepileptic drugs, including gabapentin, lamotrigine, topiramate, and others, have been marketed, but these drugs are still in the evaluation process of evidence-based medicine and are relatively expensive. CBZ remains the first-line antiepileptic treatment drug. However, as a typical BCS II drug, CBZ is generally administered orally as a solid preparation and shows erratic absorption, suboptimal bioavailability and a greatly variable plasma concentration [39]. In addition, CBZ has at least four crystal types, and the solubility and absorption rate are significantly different among these types [40]. The dissolution rate and absorption of CBZ greatly affect the blood concentration of the drug. Studies have shown that low blood concentrations may cause therapeutic failure and that elevated blood concentrations can increase the risk of toxicity. The therapeutic efficacy and serious adverse reactions of CBZ are both related to the blood concentration. Coupled with the narrow therapeutic window of CBZ (4–10 μg/mL), routine therapeutic drug monitoring (TDM) is recommended for patients taking CBZ to prevent seizures and reduce adverse events [41]. Therefore, to address these problems, we believe that administration of CBZ formulations with modified-release properties, especially delayed-release and controlled-release formulations, which can reduce the peak concentration and help to maintain the concentration within the therapeutic range, is more beneficial to patients.

In the present study, CBZ was used as the model drug, and METHOCEL* E3 premium LV hydroxypropyl methylcellulose (HPMC E3, Figure 1C), Eudragit^®^ L100-55 (L100-55, Figure 1D), and cellulose acetate (CA, Figure 1E) were optimized as the excipients. CBZ-mr-ASD with immediate-, delayed-, and controlled-release properties was successfully prepared by TFF [31,33,42,43,44,45] with HPMC E3, L100-55, and CA, defined as CBZ-ir-ASD, CBZ-dr-ASD, and CBZ-cr-ASD, respectively. CBZ-mr-ASD was then encapsulated through a dry granulation technique to prepare the corresponding CBZ-mr-ASD capsule dosage forms. The physicochemical properties of CBZ-mr-ASD and the related capsules were investigated by a wide variety of techniques. In vitro dissolution tests and an in vivo pharmacokinetic study in rats were used to illustrate the different characteristics among the three CBZ formulations.

## 2. Materials and Methods

### 2.1. Materials

CBZ (purity 99.8%), CA, HCl, magnesium stearate (MgSt), and sodium starch glycolate (EXPLOTAB^®^, SSG) were purchased from Spectrum Chemicals (Gardena, CA, USA. Chlorzoxazone (purity 98.0%) and carbamazepine-10,11-epoxide (CBZ-E, a main active metabolite of CBZ, Figure 1B, purity 99.8%) were obtained from Tokyo Chemical (Tokyo, Japan) and Sigma–Aldrich (St. Louis, MO, USA). HPMC E3 and L100-55 were generously donated by Dow Chemical Company (Midland, MI, USA) and Röhm GmbH (Darmstadt, Germany), respectively. 1,4-Dioxane and Na_3_PO_4_ were obtained from Fisher Scientific Chemicals (Fair Lawn, NJ, USA). Silicified microcrystalline cellulose (PROSOLV SMCC^®^ 90, SMCC) was purchased from JRS PHARMA LP (Patterson, NY, USA). Acetonitrile, methanol, and trifluoroacetic acid (99%), HPLC grade, were obtained from EM Industries Inc (Gibbstown, NJ, USA). Other chemicals used were analytical grade and were used as provided.

### 2.2. Preparation of CBZ-Mr-ASD

Preparations of CBZ-mr-ASD containing the model drug and different excipients (see the Supplementary Materials, Figures S1–S3) were prepared by the TFF process. Briefly, according to the compositions listed in Table 1, CBZ and the excipients were weighed accurately and dissolved in a certain volume of deionized water and/or 1,4-Dioxane and then mixed slowly to form a uniform solution by magnetic stirring. The solutions were applied onto a cryogenic solid substrate (precooled with liquid nitrogen to approximately −50 °C) of the TFF apparatus, whereby the solutions were frozen and solidified rapidly. The obtained frozen solids were collected and quickly transferred to a VirTis Advantage bench top tray freeze dryer (The VirTis Company, Inc. Gardiner, NY, USA) for lyophilization, and the temperature was gradually increased from −50 to 25 °C over 72 h. The resultant dry powders were stored in transparent vacuum desiccators at room temperature.

### 2.3. Preparation of CBZ-Mr-ASD Capsule Formulations

In this study, a dry granulation method was used to prepare the CBZ-mr-ASD capsule formulations. The desired type of CBZ-mr-ASD was mixed with SMCC90, MgSt, and SSG according to the compositions in Table 1. The well-dispersed mixtures were compressed into slug tablets with a hydraulic press (Fred S. Carver Inc., Wabash, IN, USA). The tablets were ground into granules and then sieved with 40-mesh sieves to obtain the capsule content. The content (equivalent of 30 mg CBZ) was filled into hard gelatin capsules of applicable size for further investigation.

### 2.4. Characterization of Amorphous Solid Dispersions

#### 2.4.1. Scanning Electron Microscopy (SEM)

Scanning electron microscopy (SEM) was used to examine the surface morphology of the CBZ-mr-ASD samples. Prior to imaging, the samples were adhered to aluminum stages with conductive carbon tape and then sputter-coated with gold/palladium for 60 s. SEM images were acquired using a Hitachi S-5500 field emission scanning electron microscope (Hitachi High-Technologies Corp., Tokyo, Japan) at an accelerating voltage of 10–30 kV. Digital images were captured with Quartz PCI software (Quartz Imaging Corporation, Vancouver, BC, Canada).

#### 2.4.2. Thermal Analysis

Thermal analysis was conducted by differential scanning calorimetry (DSC) coupled with a refrigerated cooling system (Model 2920, TA Instruments, New Castle, DE, USA. Approximately 10–15 mg of CBZ-mr-ASD or the related capsule formulation was loaded into aluminum pans and subsequently compressed (PerkinElmer, Waltham, MA, USA). The samples were heated at a ramp rate of 10 °C/minute from 0 to 220 °C with nitrogen as the sample purge gas at a flow rate of 40 mL/min. All data were analyzed using TA Universal Analysis 2000 Software (New Castle, DE, USA).

#### 2.4.3. X-Ray Powder Diffraction (XRPD)

CBZ-mr-ASD and the related capsule formulation were examined by wide angle X-ray powder diffraction (XRPD) using a Philips 1710 X-ray diffractometer with a copper target and nickel filter (Philips Electronic Instruments Inc., Mahwah, NJ, USA). The voltage was 40 kV, and the current was 40 mA. The samples were measured in the 2-theta range from 3 to 50° using a step size of 0.05 2-theta degree with a dwell time of 2 s.

#### 2.4.4. Hygroscopic Property

##### Moisture Property

Moisture sorption analysis was performed using dynamic vapor sorption (DVS-Intrinsic, Surface Measurement Systems Ltd., London, UK). Pre-weighed CBZ-mr-ASD or the related capsule content (5–10 mg) was loaded into quartz sample pans and equilibrated at 0% RH until a constant weight was reached. The measurements were carried out at 25 °C, and the water sorption was recorded from 0% to 90% RH in 10% increments (d*m*/d*t* ≤ 0.002 mg/min equilibrated for 10 min at each step).

##### Water Content Measurements

The residual water contents were measured by an Aquapal III Karl–Fischer Titrator (CSC Scientific Company, Inc., Fairfax, VA, USA). CBZ-mr-ASD or the related capsule formulation was accurately weighed and dissolved in anhydrous methanol and then quickly transferred to a Karl–Fischer titration vessel for titration, and the water content was calculated. Three replicates of each sample were measured with the same procedure.

#### 2.4.5. Compressibility and Fluidity Analysis

The Carr index (CI) is an important indicator for evaluating the compressibility and fluidity of powder samples. The CI was calculated from bulk density (ρ_bulk_) and tapped density (ρ_tapped_) using the equation CI = (ρ_tapped_−ρ_bulk_) × 100/ρ_tapped_ [46]. Bulk and tapped densities were measured by the cylinder method using a 50–1200 tapped density meter (Varian, Inc., Cary, NC, USA). CBZ-mr-ASD or the related capsule formulation was sieved with 18-mesh sieves (Alfa Aesar, Co., Ward Hill, MA, USA) and accurately weighed, followed by pouring into a cylinder. The volume was measured to obtain the bulk density, and then, the cylinder was tapped until the sample volume became constant to determine the tapped density.

#### 2.4.6. Drug Content Determination

An accurately weighed sample (CBZ-mr-ASD or the related capsule formulation, approximately 8 mg of CBZ) was dispersed in methanol (10 mL) and mixed thoroughly to extract CBZ. The solution was filtered through a membrane filter (0.45 μm, Gelman GHP Acrodisc, VWR, West Chester, PA, USA), and 50 μL filtrate was appropriately diluted with methanol, vortexed, and transferred to 2 mL vials (VWR International, West Chester, PA, USA) for HPLC analysis. Three replicates of each sample were measured with the same procedure, and the drug contents were expressed as percentages compared to the theoretical amount.

HPLC analysis was carried out with a Shimadzu LC-10A HPLC system (Shimadzu Corporation, Columbia, MD, USA) equipped with an Alltech Inertsil TM ODS-2 (5 μm, 150 × 4.6 mm) C_18_ column (Alltech Associates, Inc., Deerfield, IL, USA). The mobile phase consisted of water/methanol/acetonitrile (50:35:15, *V/V/V*) at a flow rate of 1.5 mL/min. The injection volume was 20 μL, the column temperature was 25 °C, and the detection wavelength was 288 nm.

#### 2.4.7. In Vitro Dissolution Testing

In vitro dissolution testing was performed according to the USP II paddle method. The paddle speed was 50 rpm, and the dissolution medium was maintained at 37 ± 0.5 °C. CBZ-mr-ASD or the related capsule sample was placed into the dissolution vessel containing 750 mL of a 0.1N HCl. After 120 min, 250 mL 0.2 M Na_3_PO_4_ solution (equilibrated to 37 ± 0.5 °C) was added to vessel to adjust the pH of the medium to 6.8. During testing, samples (0.6 mL for each) were withdrawn from vessel at predefined intervals (2, 5, 10, 20, 30, 45, 60, 120, 125, 130, 140, 150, 180, 240, 360, and 480 min) and immediately filtered with 0.45 μm filter. A 0.3 mL filtrate was diluted with 0.3 mL methanol for HPLC analysis, as in Section 2.4.6. All experiments were carried out in triplicate.

### 2.5. In Vivo Pharmacokinetic Studies

#### 2.5.1. Pharmacokinetic Studies in Rats after a Single Oral Dose

In vivo pharmacokinetic studies were carried out with jugular vein pre-catheterized male Sprague–Dawley rats (bodyweight approximately 300–350 g, Charles River Laboratories, Inc., Wilmington, MA, USA). The rats were housed individually in a 12 h light/dark cycle with free access to food and water for at least 3 days for acclimatization. Catheters were flushed every 3 days using heparinized saline (50 U/mL) to prevent clogging. These rats were divided randomly into 4 groups and fasted overnight before the experiment. Each group of rats (*n* = 6) was administered one of the formulations (crude CBZ, CBZ-ir-ASD, CBZ-dr-ASD, or CBZ-cr-ASD) at an appropriate dose of 30 mg/kg. Each formulation was filled into size 9 or 9el hard gelatin capsules (Torpac Inc., Fairfield, NJ, USA) and administered to rats using a rat capsule dosing apparatus followed by a 0.5 mL bolus of water. Blood (300 μL) was collected from the jugular vein catheter at specified time intervals (0.25, 0.5, 0.75, 1, 1.5, 2, 3, 4, 6, 8, 12, and 24 h), and each blood collection was followed by injection of an equal volume of prewarmed normal saline into the rats. The blood samples were collected into heparinized tubes, and the plasma samples were separated by centrifugation at 3000× *g* for 10 min and then stored at −80 °C until analysis.

The study protocol was approved and conducted in accordance with the Institutional Animal Care and Use Committee (IACUC) guidelines at the University of Texas in Austin. The approval number was AUP-2011-00077.

#### 2.5.2. Quantification of CBZ and CBZ-E Concentrations in Plasma

CBZ is partially metabolized to CBZ-E by the inducible hepatic cytochrome P450 enzyme, which shows antiepileptic properties similar to CBZ itself and is the main active metabolite contributing significantly to the undesirable side effects of CBZ therapy [41,47]. Therefore, a simultaneous determination of the concentrations of CBZ and CBZ-E in plasma is important for optimizing CBZ therapy on a patient-to-patient basis, as well as for studying the pharmacokinetics of CBZ and its metabolite [48,49].

The CBZ and CBZ-E concentrations in the plasma samples were quantified by HPLC, and chlorzoxazone was used as an internal standard (IS). Then, 200 μL of acetonitrile (containing IS 20 μg/mL) was added to 100 μL of the plasma sample and thoroughly vortexed for 1.0 min. Then, the mixture was centrifuged at 13000× *g* for 10 min. The supernatant was pipetted into a glass vial, and 20 μL was used for the HPLC analysis.

All analyses were performed using an UltiMate 3000 Basic Automated HPLC (Dionex Corporation, Bannockburn, IL, USA). Reverse-phase separations were achieved with an Inertsil^®^ ODS-3 C_18_ column (5 μm, 4.6 × 250 mm, GL Sciences Inc, Tokyo, Japan) with the mobile phase of methanol-acetonitrile-0.05% trifluoroacetic acid/water (27:15:58, *V*/*V*/*V*) at a flow rate of 1.2 mL/min. The column temperature was 38 °C, and the detection wavelength was 210 nm.

The pharmacokinetic parameters were calculated using a standard noncompartmental model with WinNonlin Professional Version 5.2 (Pharsight Corporation, Mountain View, CA, USA).

## 3. Results and Discussion

### 3.1. Preparation of CBZ-Mr-ASD and Related Capsule Formulations

TFF is an ultrarapid freezing process in which a homogenous solution freezes into thin films on a cryogenic surface within typically 50 to 1000 ms, depending on the properties of the solvent. The degree of supercooling is so high that nucleation and growth of crystals may be minimized or prevented, especially when the polymer is contained in the liquid feed solution. Ultrarapid freezing prevents phase separation during freezing, allowing the drug and the polymer to be molecularly dispersed within the final formulation, leading to the formation of amorphous material in the nanostructure [33,50]. The melting point of 1,4-Dioxane is approximately 12 °C, and it is miscible with water. During the TFF process, the solvent is rapidly frozen and solidified. Then the solvent is removed by sublimation during lyophilization to obtain a nanostructured matrix-like composition. Therefore, the use of 1,4-Dioxane also facilitates lyophilization, making it an ideal solvent in this study for amorphous CBZ through the TFF process. The polymers, including HPMC E3, L100-55, and CA, which have different solubilities in the GI digestive fluid, were chosen as excipients.

To obtain better physicochemical properties and increase patient compliance via an oral administration route, CBZ-mr-ASD-related capsule formulations were prepared by dry granulation tableting and grinding with SMCC, MgSt, and SSG as supporting agents.

### 3.2. Physicochemical Properties of CBZ-Mr-ASD and Related Capsule Formulations

The physicochemical properties of CBZ-mr-ASD produced by TFF and the related capsule formulations were investigated and compared to crude CBZ.

#### 3.2.1. Scanning Electron Microscopy (SEM) of CBZ-Mr-ASD

**Figure 2:**
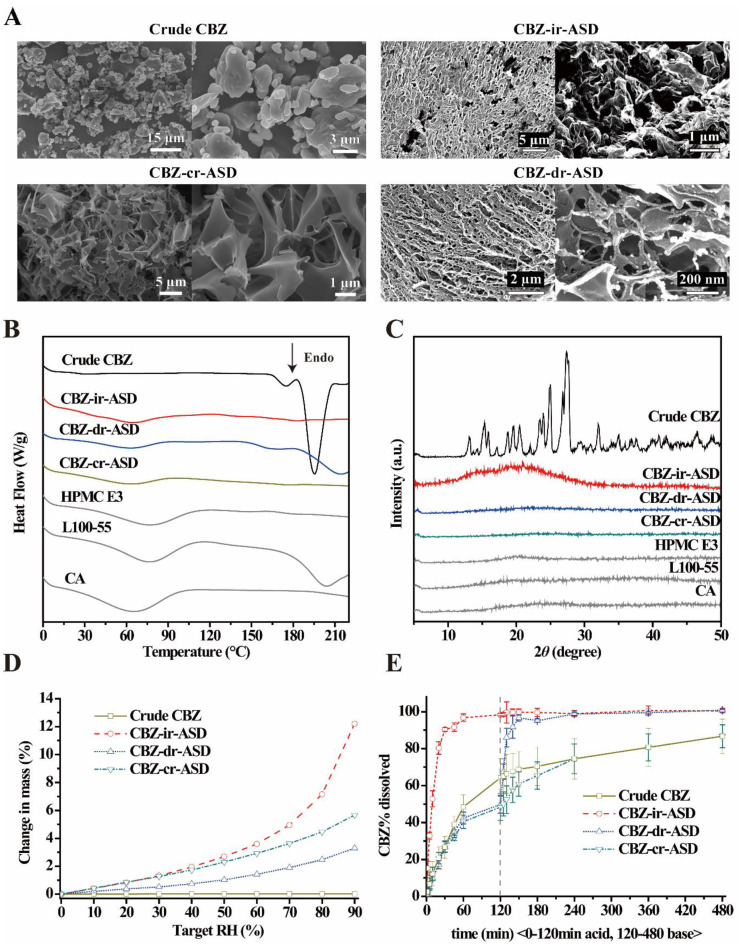
Characterization of CBZ-mr-ASD. (**A**) SEM micrographs of crude CBZ and CBZ-mr-ASD. (**B**) DSC profiles of crude CBZ, excipients, and CBZ-mr-ASD. (**C**) X-ray powder diffraction (XRPD) patterns of crude CBZ, excipients, and CBZ-mr-ASD. (**D**) Dynamic vapor sorption (DVS) isotherms of crude CBZ and CBZ-mr-ASD. (**E**) In vitro dissolution profiles of three kinds of CBZ-mr-ASD (*n* = 3).

SEM was employed to evaluate the morphology of crude CBZ and optimized CBZ-mr-ASD, and SEM images are shown in Figure 2A. The micrograph of crude CBZ showed microscale irregular, dense, and large crystal plates. In contrast, a highly porous structure with a more regular shape was observed in the micrographs of the CBZ-mr-ASD (including CBZ-ir-ASD, CBZ-mr-ASD, and CBZ-cr-ASD) (Figure 2A). Furthermore, at higher magnifications, microparticle agglomerates were loosely connected to form a sponge-like porous matrix structure, and no crystal CBZ was observed. The rapid freezing process is responsible for the homogeneous blending of drugs and polymers, yielding high-porosity nanostructured aggregates with a greater surface area, which inhibited crystal growth and significantly enhanced the dissolution rate [33,34]. Compared with the other two preparations, the structure of CBZ-ir-ASD looks more compact, which may be related to the 20% water in the solvent used in the TFF process.

#### 3.2.2. Amorphous State of CBZ-Mr-ASD

Crude CBZ, HPMC E3, L100-55, CA, and three kinds of optimized CBZ-mr-ASD were analyzed by DSC and XRPD. Heat flow thermograms are depicted in Figure 2B. Crude CBZ exhibited two obvious endothermic peaks at 173.8 and 195.3 °C, which represent the melting points of the different crystal forms of the drug and its crystalline nature. As other polymers forming ASDs, HPMC E3, L100-55, and CA possess a relatively high glass transition temperature (Tg), resulting in lower drug molecular mobility and crystallization tendency, which is more conducive to the preparation of stable ASD [51,52,53]. The characteristic endothermic peaks of CBZ disappeared in all of the CBZ-ir-ASD, CBZ-dr-ASD and CBZ-cr-ASD samples, which demonstrated the amorphous state of the drug in these dispersions. XRPD was used to detect the crystallinity of the drug. As a highly crystalline powder, crude CBZ showed distinctive peaks in the 2θ range of 13–28° and major characteristic peaks at 2θ values of 25.0 and 27.4° (Figure 2C). However, none of these correlative characteristic drug peaks could be identified in the XRD patterns of the optimized CBZ-mr-ASD samples, highlighting that the drug was completely amorphous in these three dispersions (Figure 2C), which is consistent with the DSC results.

#### 3.2.3. Hygroscopic Property

**Figure 3:**
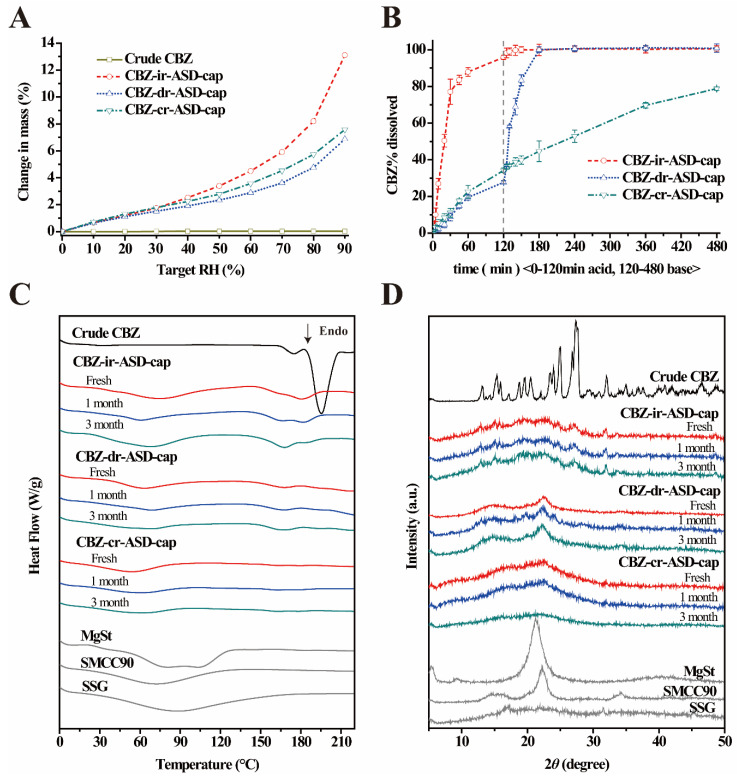
Characterization of CBZ-mr-ASD related capsule formulations. (**A**) DVS isotherm of crude CBZ and CBZ-mr-ASD capsule contents. (**B**) In vitro dissolution profiles of three kinds of CBZ-mr-ASD capsules (*n* = 3). (**C**) DSC profiles of three kinds of CBZ-mr-ASD capsules after 3 months of storage at 25 °C/30% RH. (**D**) XRPD patterns of three kinds of CBZ-mr-ASD capsules after 3 months of storage at 25 °C/30% RH.

Moisture sorption plays a key role in ASD characteristics and stability [54]. Moisture sorption can cause plasticization and increased molecular mobility, which can lead to drug crystallization [55,56]. To evaluate the properties of CBZ-mr-ASD and the related capsule formulations in the presence of moisture under stress conditions, freshly prepared samples of three kinds of CBZ-mr-ASD and the related capsule formulations were subjected to moisture sorption analysis with DVS. Representative moisture sorption isotherms are shown in Figure 2D and Figure 3A. The water uptake of the crude drug did not exceed 0.03% of the initial mass after equilibration at any given RH%. However, CBZ-mr-ASD showed continuously larger water uptake with increasing RH. Optimized CBZ-ir-ASD showed the greatest weight gain, more than 12% of the initial mass at 90% RH. CBZ-dr-ASD and CBZ-cr-ASD showed lower water uptakes of 3.29% and 5.67%, respectively. This result suggested that CBZ-ir-ASD was hygroscopic, while the other formulations were not, which should be due to the more hydrophilic nature of HPMC E3 than that of L100-55 and CA. Similar moisture sorption trends were observed for the CBZ-ir-ASD, CBZ-dr-ASD, and CBZ-cr-ASD capsule formulations, with water uptakes of 13.11%, 6.88%, and 7.58%, respectively (Figure 3A). Table 2 shows that there were very low residual water contents in fresh CBZ-mr-ASD, and the water contents of the related capsule formulations varied in the range of 0.15% to 0.95%. The CBZ-ir-ASD-related capsule formulation showed a slightly higher water content than the other samples. Hygroscopic excipients are at a higher risk for instability in the amorphous CBZ formulations and should be kept in a cool, dry place [54,55,56].

#### 3.2.4. Drug Content and Flowability

The drug content, Carr index, and flowability of CBZ-mr-ASD and the related capsule formulations are listed in Table 2. The drug contents of CBZ-ir-ASD, CBZ-dr-ASD, and CBZ-cr-ASD were 49.71 ± 0.68%, 65.62 ± 0.70%, and 33.07 ± 0.32%, respectively, which is consistent with the ratio of CBZ/excipient (described in Table 1). The drug contents of the corresponding CBZ-mr-ASD capsule formulations were slightly reduced due to the added small quantities of supporting agents during the preparation of dry granulates.

The flow properties of ASD powder are considered critical to the quality of the final formulation [57]. In general, CI < 20 represents a free flowing nature [58]. For all the CBZ-mr-ASD, the flowability was fair or passable, with CI values in the range of 16.40 to 24.67. The corresponding CI values of the CBZ-mr-ASD-related capsule formulations were much smaller (from 10.26 to 15.97), indicating that those capsule contents showed better fluidity and stronger compressibility than did CBZ-mr-ASD.

#### 3.2.5. Dissolution Testing Under Sink Conditions

In vitro dissolution testing under different pH conditions was conducted to assess the performance of the CBZ-mr-ASD samples and the related capsules. The resulting profiles and critical dissolution metrics of CBZ-ir-ASD, CBZ-dr-ASD, and CBZ-cr-ASD are shown in Figure 2E, and those of the related CBZ-mr-ASD capsules are shown in Figure 3B. For immediate-release formulations with HPMC E3, over 90% of the drug was dissolved within 120 min in the acidic phase. This dissolution was related to the great water solubility of the excipient, which provided significantly greater media dissolution rates of the drug. As expected, the dissolution of the delayed-release formulations was pH-dependent due to the enteric nature of L100-55. Poor dissolutions (<50% for the CBZ-dr-ASD and <30% for the CBZ-dr-ASD capsule) were revealed in an acidic medium of pH 1.2. However, the drug accumulation release rate from the formulations significantly increased to over 90% within 15 min following the pH change of the dissolution medium to 6.8. In contrast, 86.75% and 78.79% of the drug was sustainably released from CBZ-dr-ASD and the related capsules within 8 h, respectively, which should be attributed to the insolubility of CA in the GI digestive fluids. In general, drug release from CBZ-mr-ASD showed a similar trend as that of the related capsules, with slightly increased accumulative dissolution rates. The formation of a high-energy amorphous material can increase the predicted solubility of the drug [25,59]. Once the samples were exposed to the dissolution medium, the dissolution rates mainly depended on the solubility of the excipients [60]. The CBZ-mr-ASD-related capsules were selected for further in vivo studies to investigate the PK behaviors of the preparations with different release characteristics.

#### 3.2.6. Physical Stability Studies

The supporting agents and the contents of the freshly prepared capsules were evaluated by DSC and XRPD. As revealed in Figure 3C,D, compared to the related CBZ-mr-ASD, the drug remained in its amorphous state in all of the contents, which demonstrated that the preparation procedures should not affect the properties of these formulations with nearly unchanged main quality indexes.

A further stability study was conducted with CBZ-mr-ASD-related capsules at 25 °C/30% RH for 3 months to evaluate any changes in crystallinity. The samples were analyzed at time points of 1 and 3 months using DSC and XRPD, as described previously. The DSC results confirmed that the melting point of CBZ was absent, and the XRPD patterns exhibited no change in the peak intensity of the drug in any of the contents, even after 3 months, which indicated an essentially amorphous state of CBZ. These observations remained unchanged compared to the freshly prepared samples, confirming the ideal stability of the formulations.

### 3.3. Pharmacokinetic Studies in Rats

**Figure 4:**
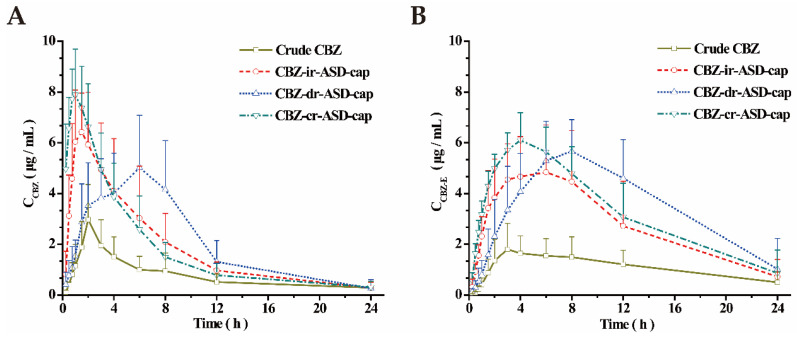
Mean plasma-drug concentrations of CBZ (**A**) and CBZ-E (**B**) in male Sprague–Dawley rats after single-dose oral administration of three kinds of CBZ-mr-ASD capsules (*n* = 6).

In this study, a modified HPLC method was further developed to enable the determination of CBZ and CBZ-E in rat plasma [61,62]. No significant interferences from endogenous plasma constituents were found in drug-free rat plasma at the retention times of the analytes and IS. The limits of detection for CBZ and CBZ-E were both 0.05 µg/mL in the present assay conditions. The peak concentration (C_max_) and time of peak concentration (T_max_) were obtained directly from the individual plasma concentration-time profiles. The area under the concentration-time curve (AUC) from zero to t (AUC_0→t_) was calculated using the trapezoidal method. The AUC can determine the bioavailability of the drug at the same dose as the formulation. The mean plasma concentration-time profiles of CBZ and CBZ-E after single-dose oral administration of each formulation in rats are presented in Figure 4, and the corresponding PK parameters are summarized in Table 3.

All the CBZ-mr-ASD capsules exhibited higher plasma concentrations of CBZ and CBZ-E than crude CBZ and maintained higher levels at each time point (Figure 4). Specifically, the C_max_ values of two analytes with three formulations were significantly higher than that of crude CBZ. For the CBZ analyte, the AUC_0-t_ values of the CBZ-ir-, CBZ-dr-, and CBZ-cr-ASD capsules were approximately 2.4-, 2.6-, and 2.5-fold greater than that of crude CBZ, respectively. For the CBZ-E analyte, the corresponding AUC_0–t_ values were approximately 2.6-, 3.2-, and 3.0-fold greater than those of the crude drug, respectively. The CBZ-mr-ASD capsules could enhance the oral absorption of the drug and improve the bioavailability of CBZ and CBZ-E, mainly due to the amorphous solid dispersion, providing a rapid increase in the concentration of the free drug in the upper GI tract. There was no significant difference in the bioavailability among the CBZ-ir-ASD, CBZ-dr-ASD, and CBZ-cr-ASD samples.

Compared to crude CBZ and the CBZ-dr-ASD formulation, the T_max_ values of CBZ-cr-ASD were increased to (5.17 ± 2.22) h for CBZ and (7.67 ± 0.82) h for CBZ-E, indicating a slower absorption and elimination of the drug with the delayed-release formulation. It was also found that the plasma concentration-time profiles of two analytes in several rats showed a slightly bimodal phenomenon after the oral administration of CBZ-dr-ASD capsules. L100-55 is an enteric excipient with pH sensitivity and dissolves at pH > 5.5. After oral administration of the CBZ-dr-ASD capsules, with the dissolution of the capsule shells, the capsule contents were released into gastric fluid. With the action of the high-efficiency disintegrating agent, water was rapidly adsorbed and dispersed within the contents, which is beneficial to the dissolution of the drug. In addition, the delayed-release formulation exhibited a larger specific surface area and higher free energy, and the drug dispersed in CBZ-dr-ASD was in its amorphous or molecular state, which is also advantageous for the dissolution of the drug. Therefore, although L100-55 was insoluble, a certain portion of the drug was dissolved in the acidic medium to cause the first absorption peak in the in vivo drug profiles. When the capsule contents entered the small intestine (pH > 5.5), the rapid solubility of the excipients caused a burst release of the remaining drug content, resulting in the second absorption peak, which was in agreement with the results of the in vitro dissolution study.

For the CBZ-ir-ASD formulation, the results showed that the drug was rapidly absorbed by the GI tract due to the high water solubility of HPMC E3. However, the CBZ-cr-ASD formulation with insoluble polymer showed a similar fast absorption compared with that of CBZ-ir-ASD. The in vitro dissolution method was a poor predictor of the release rate of the CBZ-cr-ASD capsules. This discrepancy was likely caused by various reasons. The ratio of the drug to the excipient in the CBZ-cr-ASD formulation was 1:2, which was higher than that of the other two prescriptions. The drug can be better dispersed in the excipient of a formulation with greatly reduced lattice energy and much larger specific surface area. Therefore, it was speculated that the adhesion of CBZ-cr-ASD was increased, which could prolong the contact time between the contents and the GI tract. This prolonged contact time could rapidly increase the drug concentration difference between the top layer and the basal layer of intestinal epithelial cells. These factors were all beneficial to the passive diffusion and rapid absorption of the drug. Additionally, as a result of the slower dissolution rate, CBZ-cr-ASD exhibits a lower maximum kinetic solubility in the GI tract, and additionally, the absorption of the drug is different for the various GI segments [63], ultimately leading to undesirable bioavailability. There may be other factors that have not been discovered, such as individual differences and GI digestive enzymes.

The present study demonstrated that mr-ASD prepared by TFF can be used to enhance the oral bioavailability of compounds with low dissolution rates and poor PK characteristics.

## 4. Conclusions

In our study, three kinds of CBZ-mr-ASD with immediate-, delayed-, and controlled-release properties were successfully prepared with HPMC E3, L100-55, and CA by a TFF process. The related CBZ-mr-ASD capsule formulations were further processed by a dry granulation method for convenient oral administration. The drug remained in an amorphous state in all of the preparations, and the drug dissolution rates from the CBZ-mr-ASD formulations and the related capsules were modified and enhanced compared to that of the crude drug. The bioavailability of CBZ and its main active metabolite, CBZ-E, in the CBZ-mr-ASD formulations significantly improved, accompanied with good PK profiles. Collectively, mr-ASD prepared by TFF could be a promising delivery system to enhance the oral absorption of poorly water-soluble drugs.

## Figures and Tables

**Table 1:** Compositions of CBZ-mr-ASD capsules.

Ingredients	Function	Types of CBZ-mr-ASD		
		CBZ-ir-ASD	CBZ-dr-ASD	CBZ-cr-ASD
CBZ-mr-ASD	Excipient	HPMC E3	L100-55	CA
	Ratio of CBZ/Excipient (*w*/*w*)	1:1	2:1	1:2
	Solvent	1,4-Dioxane/water (8:2, *v*/*v*)	1,4-Dioxane	1,4-Dioxane
	Solids in solution (*w*/*v*, %)	2.0	2.0	2.0
	Active and carrier	900 mg	750 mg	900 mg
SMCC90	Diluent	80 mg	200 mg	80 mg
SSG	Disintegrant	18 mg	45 mg	18 mg
MgSt	Lubricant	2 mg	5 mg	2 mg
Size		3 (60 mg)	3 (60 mg)	1 (100 mg)

**Table 2:** Drug contents, residual water contents and flow properties of CBZ-mr-ASD and the related capsule formulations (*n* = 3).

Formulation		Drug Content (%)	Residual Water Content (%)	Carr Index	Flowability *
CBZ-mr-ASD	CBZ-ir-ASD	49.71 ± 0.68	0.782 ± 0.188	19.02 ± 0.49	Fair
	CBZ-dr-ASD	65.62 ± 0.70	0.493 ± 0.059	16.40 ± 0.91	Fair
	CBZ-cr-ASD	33.07 ± 0.32	0.186 ± 0.018	24.67 ± 0.58	Passable
	CBZ-ir-ASD-cap	44.51 ± 0.20	0.574 ± 0.158	10.26 ± 1.47	Good
CBZ-mr-ASD-cap	CBZ-dr-ASD-cap	48.25 ± 0.32	0.492 ± 0.059	12.42 ± 0.55	Good
	CBZ-cr-ASD-cap	29.56 ± 0.29	0.553 ± 0.120	15.97 ± 0.25	Good

* Flowability: Evaluated by the Carr index, <10 Excellent, 11–15 Good, 16–20 Fair, 21–25 Passable, 26–31 Poor, 32–37 Very poor, >38 Extremely poor.

**Table 3:** Pharmacokinetic parameters of CBZ and CBZ-E after dosing with CBZ-mr-ASD-related capsules in rats (*n* = 6).

Parameters		T_max_ (h)	C_max_ (μg/mL)	AUC_(0–t)_ (μg/mL × h)
CBZ	Crude drug	2.0 ± 0.55	3.41 ± 0.89	18.85 ± 5.68
	CBZ-ir-ASD-cap	1.67 ± 0.75	7.38 ± 1.36	44.87 ± 16.06
	CBZ-dr-ASD-cap	5.17 ± 2.22	6.38 ± 1.01	49.63 ± 9.93
	CBZ-cr-ASD-cap	1.08 ± 0.47	8.25 ± 1.50	44.95 ± 12.22
CBZ-E	Crude drug	4.67 ± 2.66	2.24 ± 0.84	24.52 ± 10.51
	CBZ-ir-ASD-cap	5.33 ± 1.97	5.26 ± 1.76	66.88 ± 25.01
	CBZ-dr-ASD-cap	7.67 ± 0.82	6.01 ± 1.48	83.01 ± 10.68
	CBZ-cr-ASD-cap	4.17 ± 0.98	6.14 ± 1.04	78.47 ± 20.27

## Supplementary Materials

The following are available online at https://www.mdpi.com/1999-4923/12/11/1023/s1, Figure S1: The miscibility of CBZ and polymers with different ratios, Figure S2: DSC profiles of CBZ-polymer powders prepared by TFF with three different types and ratios of polymers, Figure S3: XRPD patterns of CBZ-polymer powders prepared by TFF with three different types and ratios of polymers.
