# Supplementary material for: Bioavailability Improvement of Carbamazepine via Oral Administration of Modified-Release Amorphous Solid Dispersions in Rats
Source: Pharmaceutics. 2020 Oct 26;12(11):1023. doi: 10.3390/pharmaceutics12111023 (PMC7693946; doi:10.3390/pharmaceutics12111023)
Supplement: Supplementary file 1 [file pharmaceutics-12-01023-s001.pdf]

pharmaceutics

Supplementary Materials: Bioavailability Improvement of Carbamazepine via Oral Administration of Modified-Release Amorphous Solid Dispersions in Rats

Houli Li, Meimei Zhang, Lilong Xiong, Weiyi Feng and Robert O. Williams III

1. The miscibility of CBZ and polymers

Solid mixtures consisting by CBZ and polymers with different ratios (4:1, 3:1, 2:1, 1:1, 1:2, 1:3, 1:4) were prepared by dry grinding method. Then the glass transition temperature (T*g*) of the solid mixtures were measured by DSC. Although the T*g* values of the solid mixtures could be influenced by different types and ratios of polymers, for each sample, the corresponding DSC curve only showed one single T*g* value (Figure S1). CBZ has good miscibility with HPMC E3, L100-55 and CA, and thus the three types of polymers were selected for further study.

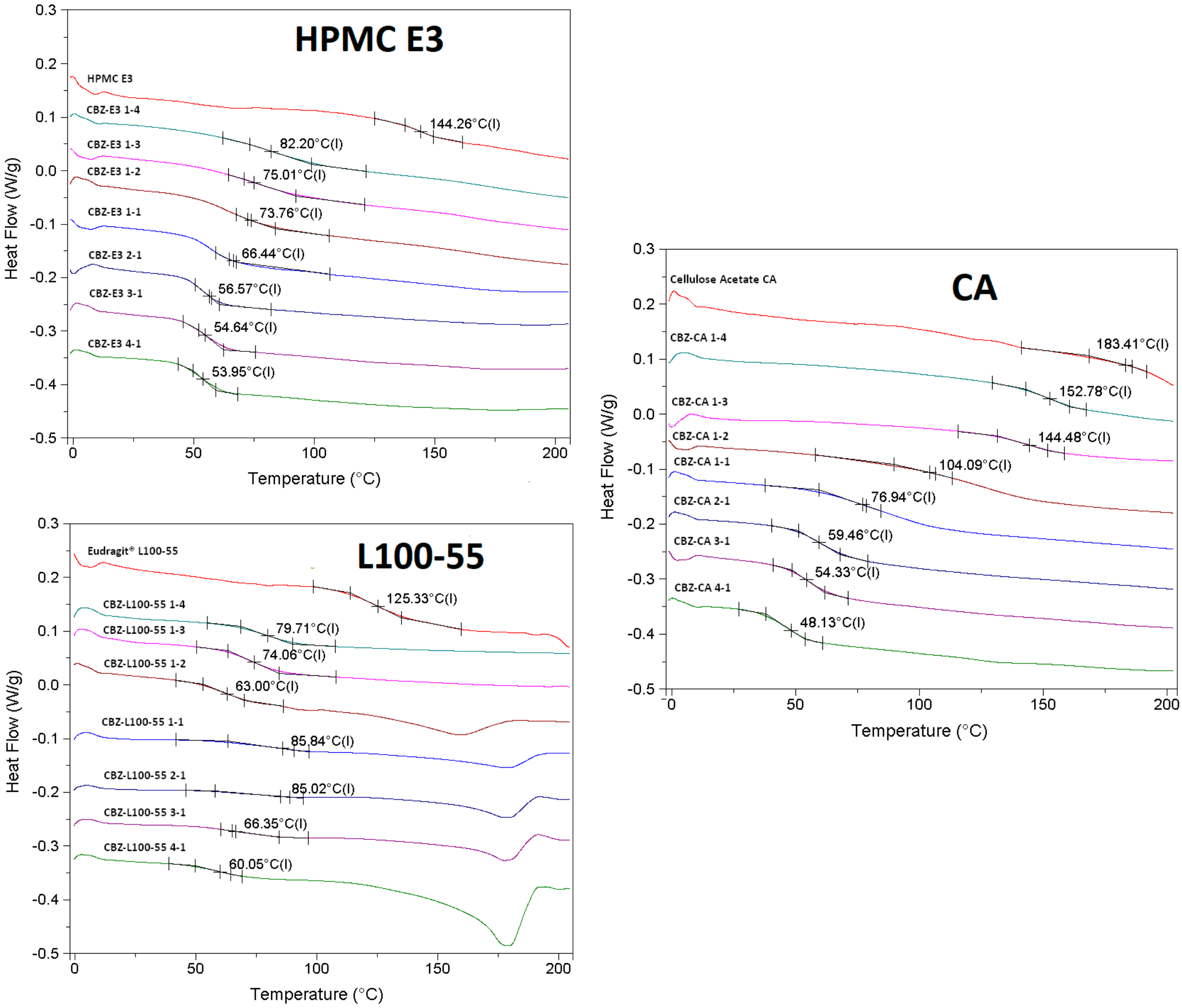

Figure S1. The miscibility of CBZ and polymers with different ratios.

2. The preparation ratio of CBZ and polymers

CBZ-polymer powders with three different polymers at various ratios (4:1, 3:1, 2:1, 1:1, 1:2, 1:3, 1:4) were successfully prepared by TFF. The properties of the powders were preliminarily investigated by DSC and XPRD. CBZ has good miscibility with HPMC E3, L100-55 and CA, however, recrystallization exotherms were also observed in some formulations (Figure S2) which revealed phase separation occurred during power formation. The XRPD results indicated that the drug was amorphous for CBZ-polymer powders with three different polymers up to a 2:1 CBZ-polymer ratio (Figure S3).

Considering the miscibility of CBZ and polymers, the T*g* value for different ratios of CBZ-polymer, the recrystallization and the amorphous state of the drug, etc., the CBZ-polymer ratios for HPMC E3, L100-55 and CA were selected as 1:1, 2:1 and 1:2, respectively.

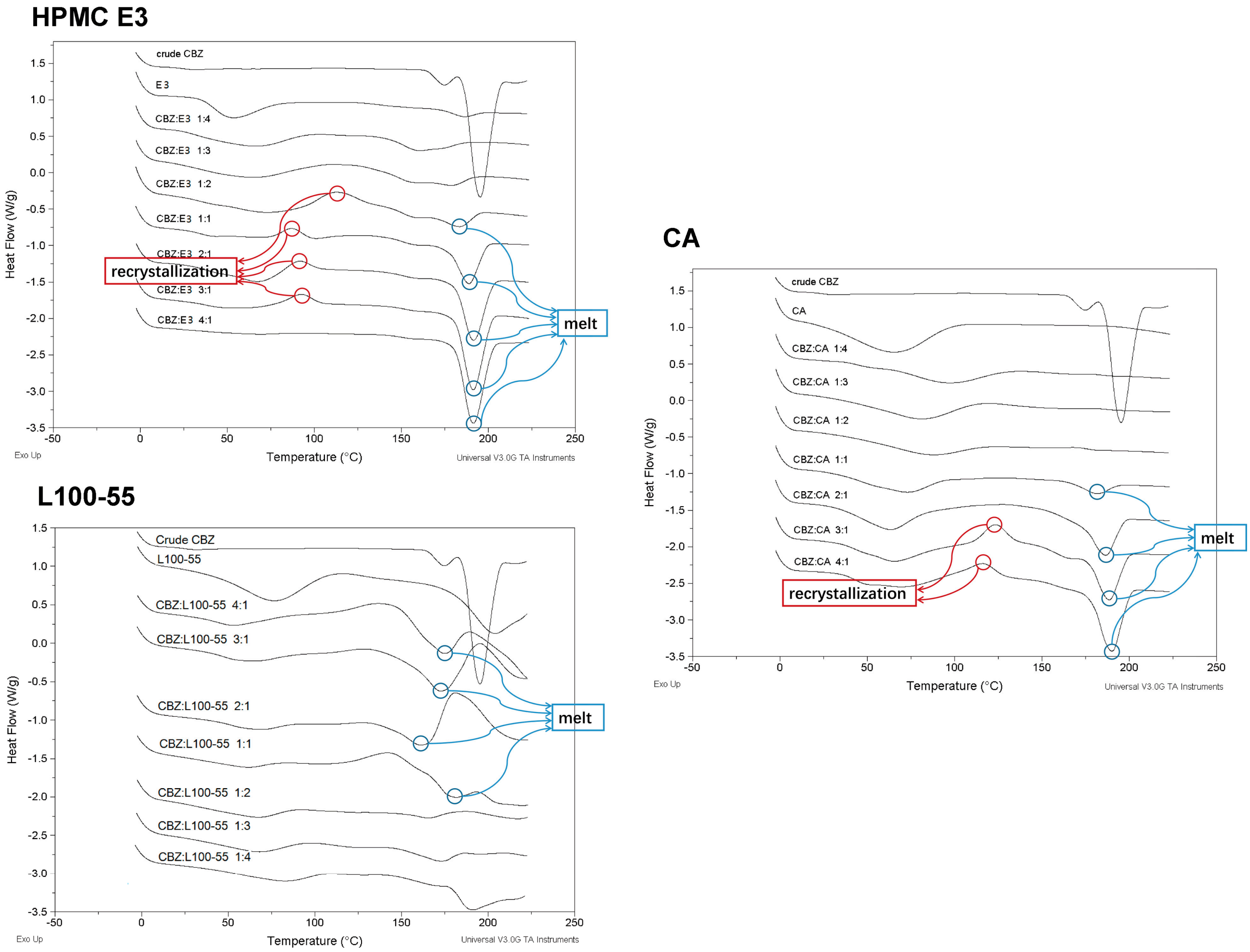

Figure S2. DSC profiles of CBZ-polymer powders prepared by TFF with three different types and ratios of polymers.

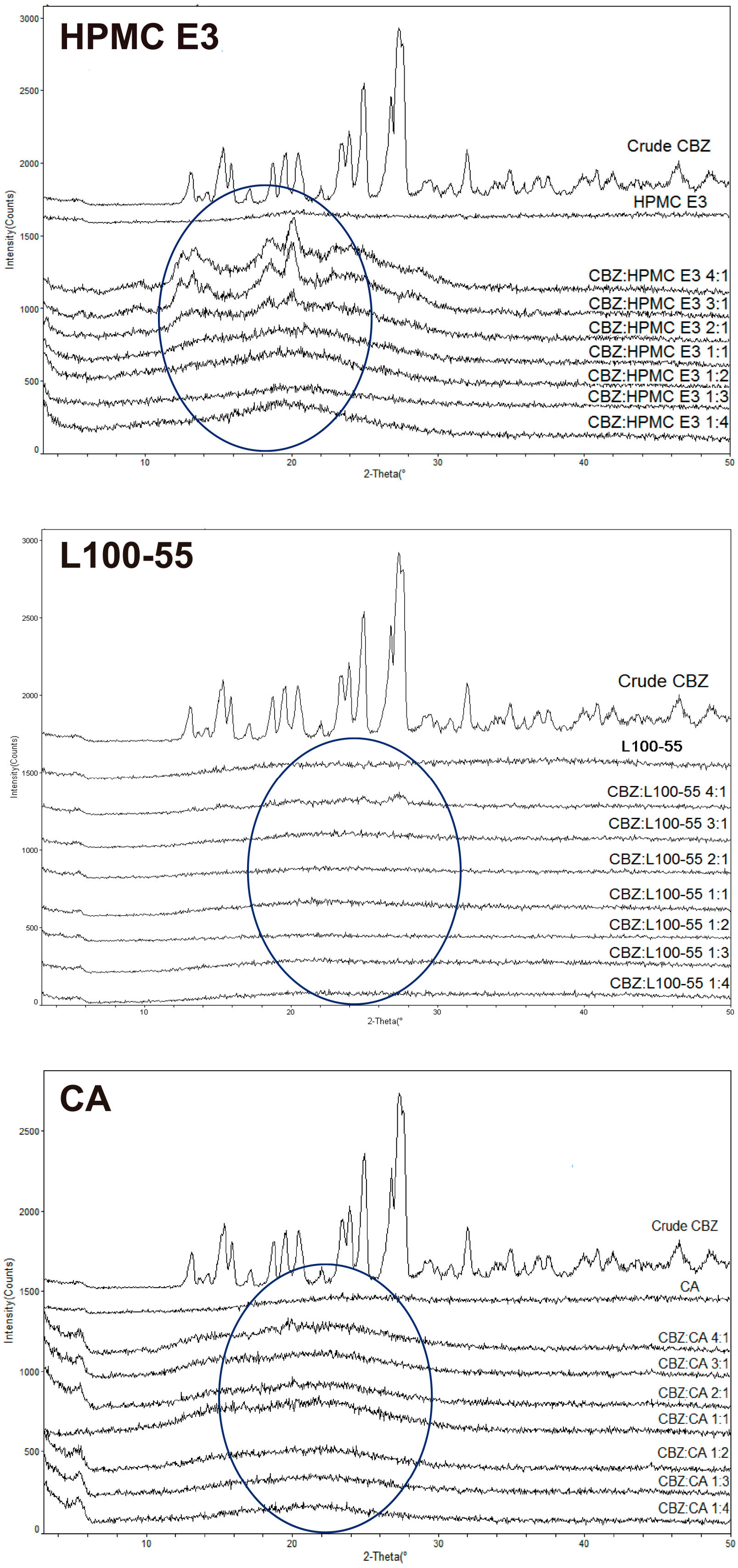

Figure S3. XRPD patterns of CBZ-polymer powders prepared by TFF with three different types and ratios of polymers.
